# Investigation of mechanism: spoof SPPs on periodically textured metal surface with pyramidal grooves

**DOI:** 10.1038/srep32008

**Published:** 2016-08-25

**Authors:** Lili Tian, Jianlong Liu, Keya Zhou, Yang Gao, Shutian Liu

**Affiliations:** 1 Harbin Institute of Technology, Department of Physics, Harbin, 150001, China; 2 Heilongjiang University, College of Electronic Engineering, Heilongjiang, 150080, China

## Abstract

In microwave and terahertz frequency band, a textured metal surface can support spoof surface plasmon polaritons (SSPPs). In this paper, we explore a SSPPs waveguide composed of a metal block with pyramidal grooves. Under the deep subwavelength condition, theoretical formulas for calculation of dispersion relations are derived based on the modal expansion method (MEM). Using the obtained formulas, a general analysis is given about the properties of the SSPPs in the waveguides with upright and downward pyramidal grooves. It is demonstrated that the SSPPs waveguides with upright pyramidal grooves give better field-confinement. Numerical simulations are used to check the theoretical analysis and show good agreement with the analytical results. In addition, the group velocity of the SSPPs propagating along the waveguide is explored and two structures are designed to show how to trap the SSPPs on the metal surface. The calculation methodology provided in this paper can also be used to deal with the SSPPs waveguides with irregular grooves.

In optical frequency band, a smooth dielectric-metal interface can sustain surface plasmon polaritons (SPPs), which can propagate along the surface with high field-confinement. In microwave and terahertz frequency range, the metal behaves like a perfect electrical conductor (PEC) and SPPs cannot be confined on a smooth metal surface. However, a textured dielectric-metal interface can bound another kind of surface electromagnetic waves, which are called spoof SPPs (SSPPs)^1^,^2^,^3^. The dispersion and electromagnetic distributions of SSPPs are similar to those of the conventional SPPs. It offers fascinating possibility of controlling terahertz wave and microwave with high field-confinement on metal surface. Like its analogues in optical frequency range, the properties of SSPPs in microwave or terahertz band are also sensitive to the geometric shape of the metal surface^3^,^4^,^5^,^6^,^7^,^8^, which makes it possible to realize novel functional devices by modulating the structure geometry. In the past ten years, the dispersion, excitation and propagation of the SSPPs have been investigated extensively^3^,^5^,^6^,^7^,^8^,^9^. Due to their intriguing properties and the potential applications in many areas^10^,^11^,^12^,^13^, SSPPs have become one of the hotspots in recent researches.

A metal block drilled with one-dimensional periodic grooves on the surface is one representative among various SSPPs waveguides^3^,^5^,^6^. It has been demonstrated that the properties of the SSPPs supported by this kind of waveguide is insensitive to the waveguide thickness, which provides great flexibility and practicability in fabrication^14^,^15^,^16^,^17^. In previous studies, SSPPs waveguides with rectangular grooves are the first to be realized due to its geometric simplicity. Afterwards, waveguides with trapezoidal, V-shaped, slanted rectangular and half-moon grooves have been proposed and studied both in theory and experiment. It has been shown that the shape of the groove has apparent influence on the field-confinement of SSPPs^6^,^18^,^19^,^20^. However, to our knowledge, there still lacks a unified theoretical description about what kinds of SSPPs waveguide will give a better field-confinement. In this paper, we propose a general model and formulas to calculate the dispersion relation of the SSPPs waveguide with pyramidal grooves. Rectangular groove is just one special case. Based on this model, the propagation constant and the field-confinement properties can be deduced readily. Such method can be extended to SSPPs waveguides with irregular grooves.

## Results

### Waveguides with periodic pyramidal grooves

**Figure 1:**
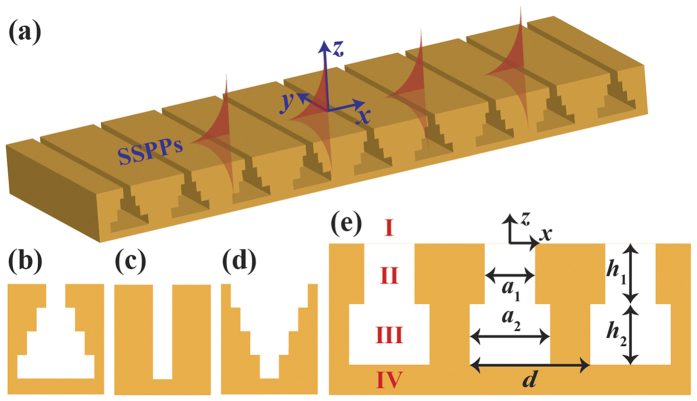
(**a**) The sketch of the SSPPs waveguide with periodic pyramidal grooves that consist of *N* rectangular grooves. (**b**–**d**) Front views of a unit cell of the waveguides with upright pyramidal grooves (**b**), rectangular grooves (**c**) and downward pyramidal grooves (**d**). (**e**) The waveguide sketch when *N* = 2.

Figure 1(a) shows the sketch of the waveguide corrugated with periodic pyramidal grooves. In this paper, we only consider the two-dimensional case where the waveguide is infinite in *y* direction. Figure 1(b) is the front view of a unit cell of the waveguide. The period constant is *d*. Each pyramidal groove can be divided into a group of slim rectangular grooves which are stacked sequentially. The number of the rectangular grooves is denoted by *N (N* = 4 in Fig. 1(a)). Correspondingly, the groove widths and depths are set as *a*_*i*_ and *h*_*i*_ (*i* = 1, 2, 3, …, *N*), respectively. In this paper, we regard the pyramidal groove to be upright or downward if its width increases or decreases gradually from top to bottom. If they are equal to each other, the pyramidal groove becomes a rectangular groove. These three types of pyramidal grooves are displayed in Fig. 1(b–d), respectively. The SSPPs waveguide with rectangular grooves has been studied extensively^3^,^5^,^21^,^22^. Under the deep subwavelength condition *λ*_0_ ≫ *d* > *a (λ*_0_ is the wavelength in free space, *a* is the groove width), the dispersion relation of the SSPPs is expressed as 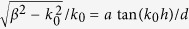
3,5,22. As shown in Fig. 1(b–d), such waveguide is just a special case of the waveguides with pyramidal grooves. In the following parts, we will give a more general analysis on the dispersion relations of the SSPPs for all these types of waveguides. Our derivation is under the PEC approximation.

### Dispersion relation for the SSPPs waveguide when *N* = 2

First, we consider the case that the pyramidal groove is composed of two rectangular grooves, as depicted in Fig. 1(e). For the upright and downward pyramidal groove, the rectangular groove widths satisfy *a*_1_ < *a*_2_ and *a*_1_ > *a*_2_, respectively. It is convenient to divide the space into four regions (labeled as I, II, III and IV, respectively). The electromagnetic field of the SSPPs propagating along *x* direction satisfies 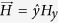 and 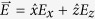. According to the MEM, the magnetic-field component *H*_*y*_ in regions I, II, III and IV can be written as


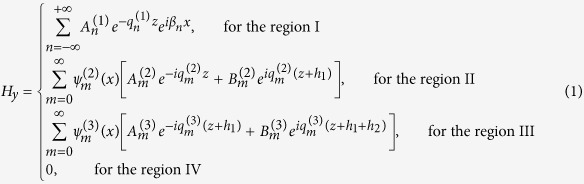


where *β*_*n*_ = 2π*n*/*d* (|*β*| ≤ π/*d*) are the propagation constants and 
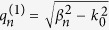
 are wave vectors in *z* direction. *n* are integers and denote the diffraction orders. 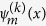 (*k* = 2, 3) correspond to the eigen-functions along *x*-direction within the rectangular grooves in regions II and III, given by


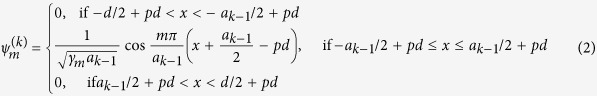


where 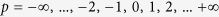. *γ*_*m*_ are constants associated with the mode number *m*. 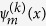 satisfy the orthogonality 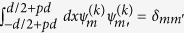. Thus *γ*_*m*_ are equal to 1 if *m* = 0 and 1/2 if *m* ≠ 0. The wave vectors 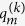 in regions II and III can be expressed as 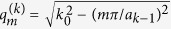.

The electric field component in the four regions can be obtained straight forwardly through 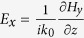 as follows,


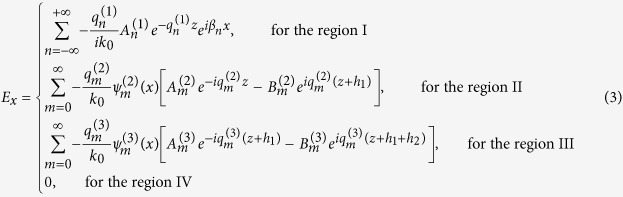


Both *H*_*y*_ and *E*_*x*_ must satisfy the boundary condition of electromagnetic fields. Noting that *H*_*y*_ are not continuous at the bottoms of the grooves, we obtain an equation set composed of five equations by applying the continuity condition at the interface *z* = 0, −*h*_1_ and −(*h*_1_ + *h*_2_). The condition that makes the equations set solvable can yield the dispersion relation for the waveguide. The dispersion formulas we obtain are of very complicated forms and they are different for the waveguides with *a*_1_ < *a*_2_ and *a*_1_ > *a*_2_. Like the waveguide with rectangular grooves^3^,^22^,^24^, we use the deep subwavelength condition *λ*_0 _≫ *d* > *a*_*k−*1_ to simplify the formulas, under which the high-order modes of electromagnetic expansions in the three regions can be neglected. In this case, the dispersion relations have the same form for the cases of *a*_1_ < *a*_2_ and *a*_1_ > *a*_2_, which is written as


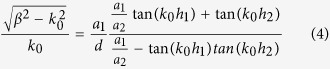


Obviously, when *a*_1_ = *a*_2_, equation (4) is simplified to


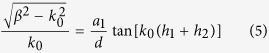


and when *a*_2_ = 0, it reduces further to


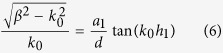


Equations (5) and (6) are dispersion relations for waveguides with rectangular grooves whose depths are *h*_1_ + *h*_2_ or *h*_1_, respectively.

From equation (4), we can see that the propagation constants *β* of the SSPPs sustained by the waveguide with pyramidal grooves are decided by the groove widths (*a*_1_, *a*_2_) and depths (*h*_1_, *h*_2_). When the groove depth *h (h* = *h*_1_ + *h*_2_) is small, only a fundamental SSPPs mode can exist. If the groove is deep enough, high-order SSPPs modes will appear. Each SSPPs mode corresponds to a frequency band that does not overlap with the others. For the fundamental mode, we can deduce from equation (4) that *β* increases with the increasing of *a*_2_ and decreasing of *a*_1_. As a consequence, the waveguide with upright pyramidal grooves has a larger propagation constant *β* than that of the downward pyramidal grooves with reversed widths. The values of *β* for the case of rectangular grooves (*a*_1_ = *a*_2_) will fall in between the other two types. From equation (1), it can be deduced that larger propagation constant *β* means higher field-confinement in *z* direction. Namely, waveguides with upright pyramidal grooves has better field-confinement than those with downward pyramidal grooves. As the case becomes different and complicated when considering high-order modes, we will focus our discussions on the fundamental mode in this paper.

**Figure 2:**
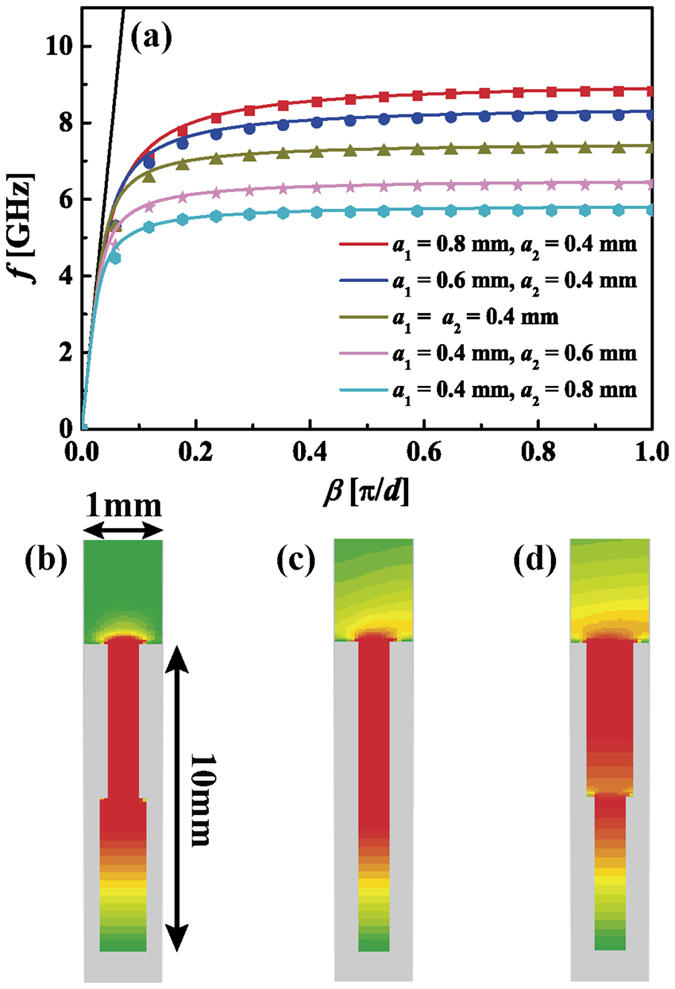
(**a**) Dispersion curves of SSPPs sustained by waveguides with pyramidal grooves that consist of two rectangular grooves. The curves and symbols represent analytical and simulated results, respectively. (**b**–**d**) The corresponding *E*_*x*_ distributions of the SSPPs on the *xz* planes. (**b**) *a*_1_ = 0.4 mm, *a*_2_ = 0.6 mm, *f* = 6.38 GHz, *β* = π/*d*. (**c**) *a*_1_ = *a*_2_ = 0.4 mm, *f* = 6.84 GHz, *β* = 0.176π/*d*. (**d**) *a*_1_ = 0.6 mm, *a*_2_ = 0.4 mm, *f* = 6.90 GHz, *β* = 0.118π/*d*.

To confirm the theoretical analysis above, we give some examples and compare with the simulated results. In all designs, the period *d* is set to be 1 mm. The depth of the pyramidal groove is fixed at *h* = *h*_1_ + *h*_2_ = 10*d* to guarantee the deep subwavelength condition^23^,^24^. For convenience, we assume *h*_1_ = *h*_2_. Five sets of (*a*_1_, *a*_2_), (0.4 mm, 0.8 mm), (0.4 mm, 0.6 mm), (0.4 mm, 0.4 mm), (0.4 mm, 0.6 mm) and (0.4 mm, 0.8 mm), are taken for groove widths. The simulated dispersion relations are calculated using finite integration method. To make it more practical, we set the waveguide thickness to be 20*d* instead of infinity in the simulation. It has been demonstrated that the dispersion relations hardly change when the thickness is larger than 20*d*^16^. In Fig. 2(a), the black line is the light line, which represents the dispersion relation of light in the free space. The curves and symbols correspond to the analytical and simulated results, respectively. It can be seen that our analytical solutions are in good agreement with the simulated results. And the dispersion curves for *a*_1_ < *a*_2_ are always lower than those for *a*_1_ > *a*_2_, which is consistent with our theoretical analysis.

Figure 2(b–d) depict the simulated *E*_*x*_ distributions of SSPPs on three different waveguides whose (*a*_1_, *a*_2_) are (0.4 mm, 0.6 mm), (0.4 mm, 0.4 mm) and (0.6 mm, 0.4 mm), respectively. The three *E*_*x*_ distributions are evaluated at 6.38 GHz, 6.84 GHz and 6.90 GHz. The corresponding propagation constants are π/*d*, 0.176π/*d* and 0.118π/*d*, respectively. The propagation constant remarkably increases when the mouth of the groove shrinks. The field distributions in Fig. 2(b–d) also demonstrate the fact that the waveguides with upright pyramidal grooves exhibit better field confinement.

### Dispersion relation for the SSPPs waveguide when *N* > 2

For the SSPPs waveguides drilled with pyramidal grooves that have more than two layers, the dispersion relation of the SSPPs can also be obtained by using the MEM. The direct derivation could be very complicated. In this paper, we utilize an analogy method. Comparing equations (4) and (6), we find that equation (4) can be obtained by replacing 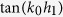 in equation (6) with


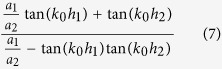


It indicates that the two-layer groove is equivalent to a single-layer groove whose width is *a*_1_ and its depth *h*_1,2_ satisfies


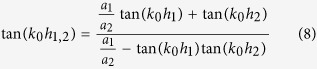


Based on this thought, the theoretical formulas for the waveguides with *N* = 2 can be extended to those with *N* > 2. First, we use an equivalent rectangular groove to replace the *N*^th^ and (*N*−1)^th^ layers. The width and depth of the equivalent groove are 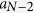 and 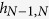 which satisfies


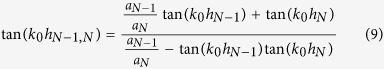


Then the (*N* − 2)^th^ layer and the equivalent layer could be replaced by a new equivalent groove whose width and depth are denoted as 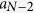 and 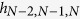 that satisfy


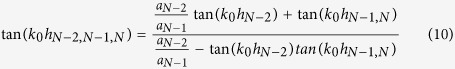


and so forth. Finally, the pyramidal groove with *N* layers could be equivalent to a single-layer rectangular groove. By this way, we can obtain the analytical dispersion relations for the waveguides with pyramidal grooves.

**Figure 3:**
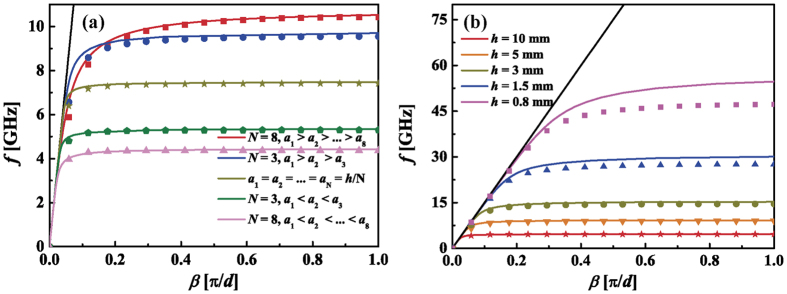
(**a**) The dispersion curves of SSPPs supported by the waveguides with pyramidal grooves that have *N (N* > 2) layers. (**b**) Dispersion relations for the waveguides with upright pyramidal grooves which have 6 layers as the groove depth varies. The groove depth varies from 10*d* to 0.8*d*. The curves and symbols correspond to analytical and simulated results, respectively.

Likewise, we use the simulated results to verify such analogy. The period is still fixed at *d* = 1 mm. The groove depth of each rectangle groove layer is equal to the others, i. e., *h*_1_ = *h*_2_ = … = *h*_*N*_ = *h*/*N (h* = 10*d*). For the waveguide with upright pyramidal grooves, the width of the top rectangular groove is set as 0.1 mm. The widths of the rectangular grooves below increase evenly with a step of 0.1 mm. The waveguide with downward pyramidal grooves is just the opposite. Figure. 3(a) shows the analytical and simulated results when *N* = 3 and *N* = 8. It is easy to identify that the analytical results match the simulated results well, which validates our analogy. Still, the field-confinement of the waveguide with upright pyramidal grooves is better than that with downward pyramidal grooves. Additionally, as the number of the layers increases, the field-confinement of the waveguide with upright pyramidal grooves gets better, while the field-confinement of its counterpart becomes weaker. The field-confinement properties for the different types of grooves can be easily understood if we equate the multi-layered groove to a single straight groove. The equivalent depth for the upright pyramidal groove is obviously larger than its counterpart. The deeper rectangular grooves correspond to higher field-confinement. Therefore, the waveguide with upright pyramidal grooves is of the highest field-confinement.

All the waveguides discussed above have the same groove depth that decuples the period. In this case, the analytical and simulated results for the fundamental modes are in complete agreement. However, as groove depths of the waveguides decrease, the eigen-frequency will increase and the error brought by the deep subwavelength approximation will grow. In fig. 3(b), dispersion relations for five waveguides with upright pyramidal grooves that have 6 layers are given to show how the deviation changes with the groove depths. Their structure parameters are set as the waveguides discussed above except the groove depths. Figure 3(b) shows the analytical and simulated results when the groove depth takes 10*d*, 5*d*, 3*d*, 1.5*d* and 0.8*d*. It can be seen that the analytical results move away from the simulated results gradually as the groove depths decrease. Fortunately, the differences between the analytical and simulated results are always within an acceptable range even when the depth is smaller than the period. It suggests that our theoretical formulas can be used to describe the characteristics of the waveguide with a proper precision.

**Figure 4:**
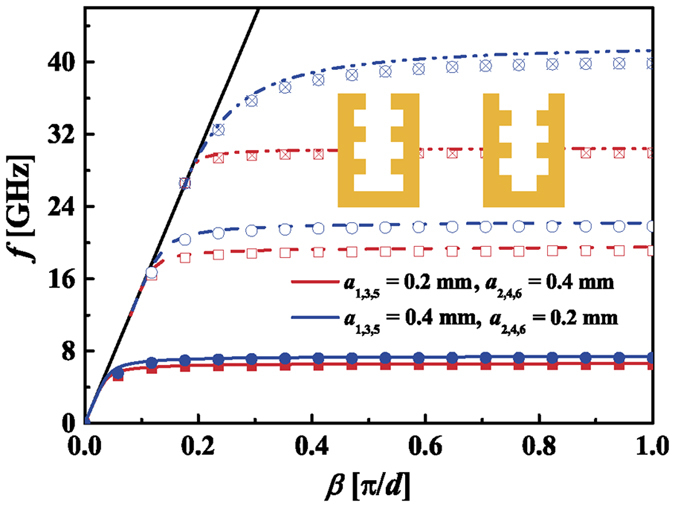
The dispersion curves of SSPPs in the waveguide with irregular pyramidal grooves that have 6 layers. The solid, dash and dash dot curves denote the fundamental, the 1^st^ and the 2^nd^ order SSPPs modes, respectively. The curves and symbols correspond to analytical and simulated results, respectively. Insets: The period unit of the waveguide with irregular pyramidal grooves.

Figure 4 depicts the dispersion relations of two waveguides with irregular pyramidal grooves that have 6 layers. The waveguide corresponds to the red curve is of *a*_1,3,5_ = 0.2 mm and *a*_2,4,6_ = 0.4 mm. The other has the widths *a*_1,3,5_ = 0.4 mm and *a*_2,4,6_ = 0.2 mm. The period cells of the two waveguides are shown in the insets. We can see that the analytical results are consistent with the simulated results. It indicates that our theoretical formulas are applicable to waveguides with multi-layer rectangular grooves whose widths do not change evenly. To verify the application of our formulas to high-order SSPPs modes, we also illustrate the dispersion curves for the 1^st^ and 2^nd^ modes in Fig. 4. They are denoted by the dash and dash dot curves, respectively. It can be easily observed that our formulas are applied to the high-order modes. As the frequency increases, the difference between the analytical and simulated results grows. This is because the error brought by the deep subwavelength approximation becomes larger.

### Dispersion relations for waveguides with trapezoidal and slanted grooves

For the waveguide where the width of the grooves changes continuously from top to bottom, it is hard to get a single formula for the dispersion relation. An alternate method is to divide the groove into stacked slim rectangular layers and then calculate the dispersion relation using the analogy methods provided above. Two examples are given to check the validity of the proposed method.

**Figure 5:**
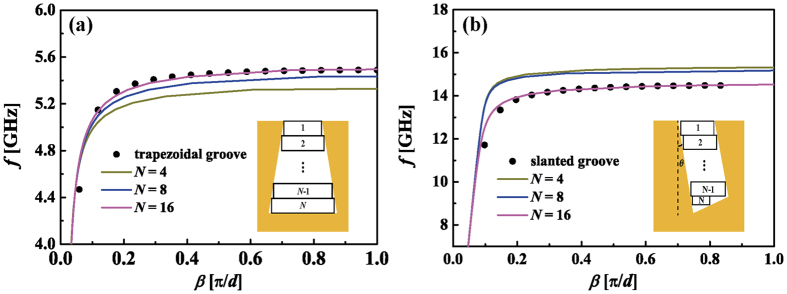
(**a**) Dispersion relations of the waveguide with trapezoidal grooves and their equivalent waveguides. (**b**) Dispersion relations of the waveguide with slanted grooves and their equivalent waveguides. The symbols represent the simulated results for the waveguide with trapezoidal and slanted grooves. The curves denote the analytical results for the equivalent waveguides.

The first one is a SSPPs waveguide with upright trapezoidal grooves^18^,^19^ whose top and bottom widths are *a*_*t*_ = 0.2 mm and *b*_*t*_ = 0.8 mm, respectively. The second one is textured with slanted grooves^6^ whose width takes *a*_*s*_ = 0.2 mm. The orientation angle is denoted with *θ*, which takes 10° here. Both waveguides have a period constant *d* = 1 mm and the groove depths are *h*_*t*_ = 10*d* and *h*_s_ = 5*d*, respectively. First, we use numerical simulation to get the dispersion relations of the SSPPs modes. The simulated results are denoted by symbols in Fig. 5. Then, we use the analogy method to get the analytical results. We divide the trapezoidal and slanted groove into *N* slim rectangular layers, as shown in the insets of Fig. 5(a,b). For the trapezoidal grooves, each layer has the same groove depth *h*_*t*_/*N*. The width of the top rectangular groove is *a*_*t*_. The widths below increase evenly with a step of (*b*_*t*_*−a*_*t*_)/*N*. For the slanted groove, the width and depth of the *N*^ th^ rectangular groove are 0.5*a*_*s*_/cosθ and 0.5*a*_*s*_sinθ, respectively. The rectangular layers above have the same width and depth, which are obtained through


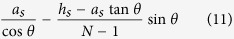


and


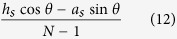


respectively. The dispersion relations for the equivalent waveguides are calculated using the theoretical formulas. The curves in Fig. 5 depict our analytical results as *N* takes 4, 8 and 16. It can be seen that the analytical results approach to the simulation results gradually as the layer number increases. Specially, when *N* = 16, the two results are nearly the same. It indicates that the dispersion relations for the waveguides with continuously changed grooves can be described by stacked layered grooves and can be calculated using the analogy method. This also explains why the dispersion curves of the waveguides with rectangular grooves lie between those with upright and downward trapezoidal grooves^18^,^19^.

### A new method to trap SSPPs

When SSPPs propagate along the waveguide drilled with grooves, their group velocity *v*_*g*_, given by *v*_*g*_ = *dω*/*dβ (ω* is the angular frequency), is less than the speed of light. As illustrated in Fig. 2(a), each dispersion curve has an asymptotic frequency, at which the group velocity becomes zero. The asymptotic frequency varies with the parameters of grooves. This feature can be utilized to realize SSPPs trapping on the metal surface.

The group velocity derived from the equation (4) is written as


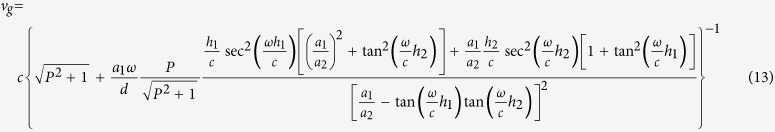


where


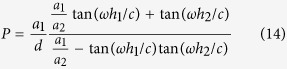


**Figure 6:**
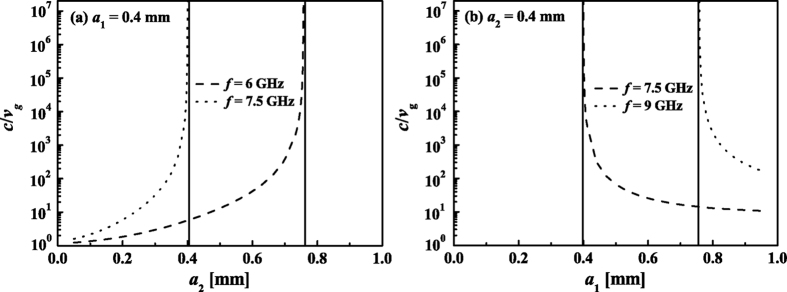
(**a**) *c*/*v*_*g*_ changing with *a*_2_ when *a*_1_ = 0.4 mm. (**b**) *c*/*v*_*g*_ changing with *a*_1_ when *a*_2_ = 0.4 mm.

We still set *d* = 1 mm, *h* = 10*d* and *h*_1_ = *h*_2_. We consider two cases here. In the first case, *a*_1_ is fixed at 0.4 mm. We calculate the group velocity *v*_*g*_ as *a*_2_ increases from 0.1 mm using equation (13). The results are displayed in Fig. 6(a). We can see that there has an asymptotic width for each frequency. When *a*_2_ approaches to the asymptotic width, *v*_*g*_ tends to zero. The higher frequency corresponds to a smaller asymptotic width. In the second case, we fix *a*_2_ at 0.4 mm and calculate *v*_*g*_ as *a*_1_ decreases from 0.9 mm using equation (13). The results shown in Fig. 6(b) indicate that the higher frequency corresponds to larger asymptotic width, which is opposite to the first case.

**Figure 7:**
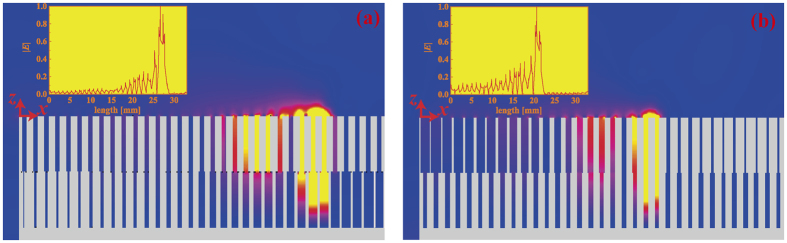
The simulated field distributions of the slow-wave structures at (**a**) *f* = 6 GHz and (**b**) *f* = 7.5 GHz. Insets: The one-dimensional |*E*| distributions along *x* direction 0.01 mm above the waveguide surface.

Utilizing the changing of the group velocity with groove widths, we design two waveguides to trap SSPPs, as shown in Fig. 7. Both of the two waveguides have 33 periods. Their period constant and groove depth are the same as above. The thicknesses of the waveguides take 20*d*. The right waveguide has fixed *a*_1_ = 0.4 mm, while its *a*_2_ increases evenly from 0.1 mm to 0.9 mm. The left one has constant *a*_2_ = 0.4 mm, while its *a*_1_ decreases evenly from 0.9 mm to 0.1 mm. We use the finite integration method to simulate the propagation of SSPPs. A plane wave propagating in *x* direction serves as the wave source. Figure 7(a,b) show the simulated electric field (|*E*|) at 6 GHz and 7.5 GHz, respectively. The insets depict the one-dimensional |*E*| distributions along *x* direction 0.01 mm above the waveguide surface. It is easy to identify that the light is trapped at the groove where the group velocity approaches zero, which is consistent with the results in Fig. 6.

## Discussion

We have studied the waveguides corrugated with pyramidal grooves and provided a general method to calculate the dispersion relations for the waveguides with various types of grooves. According to the theoretical formulas we obtained, the width and depth of the rectangular groove in each layer can determine the dispersion relations. This explains why the groove shapes can influence the field-confinement of the SSPPs. Even though our theoretical formulas are derived for the waveguide with pyramidal grooves, it can be applied to waveguides with irregular grooves since a groove in any shape can be divided into slim layers. In addition, the formulas are also applied to high-order SSPPs modes.

It is worth to note that these conclusions above are based on the deep subwavelength approximation. For waveguides that do not satisfy the condition *λ*_0_ ≫ *d*, the dispersion relations obtained by our theoretical formulas have some deviation with the exact results. Fortunately, the deviation is within an acceptable range. The approximate dispersion relation formulas can always be used to explore the characteristics of the waveguide.

Another issue is that the metal is assumed as a PEC in the analysis for simplicity. Practically, we have to take into account the finite conductivity of the waveguide. In this case, the propagation constant of SSPPs becomes a complex value. Its real and imaginary parts represent propagation and loss, respectively. It has been demonstrated that the real part is close to that under the PEC approximation^5^, while the loss grows as the field-confinement increases^25^. In many cases, this part of loss is tolerable comparing with the superiority of the high field-confinement. The SSPPs can still propagate a long distance along the metal waveguide^15^,^16^. Thus, the PEC approximation has been widely used in theoretical analysis and numerical simulation.

For the waveguide drilled with rectangular grooves, the dispersion relations, especially the asymptotic frequencies, are not sensitive to the groove width. Therefore, the rainbow-trapping effect in waveguides is always realized by changing the groove depths in previous literatures^26^,^27^,^28^,^29^. Our research results demonstrate that the dispersion relation of the SSPPs for the waveguide with pyramidal grooves can be modulated by the widths of grooves. It offers a new method to realize rainbow-trapping.

## Methods

### Model expansion method (MEM)

The MEM is a numerical method to solve the Maxwell’s equations. It is widely used to deal with the transmission and scattering problems of the electromagnetic waves in the waveguides and photonic crystals. The main idea of the MEM is as follows. First, we use the Maxwell’s equations to find a set of complete functions in each region of the scattering structure. Then we use the superposition of these functions to meet the corresponding boundary conditions of scattering field and establish the coupling equations. Finally, we solve the coupled equations to get the expansion coefficient of each region, which represent the information related to scattering. In this paper, we use the MEM for modes analysis and do not solve the expansion coefficient of each region. The dispersion formulas of the propagation modes can be obtained through the condition that makes the continuity condition at work.

### Analogy method

Analogy is a process of transferring information or meaning from a particular subject to another. In a narrower sense, analogy is an inference from one particular to another particular, which is opposed to deduction, induction and abduction. In this paper, we use the analogy method to get the dispersion formulas for the waveguide with pyramidal grooves that consist of multi-layer rectangular grooves. This method avoids complicated formulas derivation.

### Simulation

The simulated dispersion relations and field distributions of the SSPPs are obtained with the finite integration method. In the simulation, for dispersion relations, the metal was treated as a perfect electric conductor (PEC) and only a period cell is used. In the periodic direction, the “periodic” boundary condition is used. While in the other directions, we use the “electric” boundary conditions. Eigen-mode Solver is used for solving. In the simulation for field distributions, we use the boundary condition of “open (add space)” and the Time Domain Solver.

## Additional Information

**How to cite this article**: Tian, L. *et al*. Investigation of mechanism: spoof SPPs on periodically textured metal surface with pyramidal grooves. *Sci. Rep.* **6**, 32008; doi: 10.1038/srep32008 (2016).
